# Seasonal Genetic Influence on Serum 25-Hydroxyvitamin D Levels: A Twin Study

**DOI:** 10.1371/journal.pone.0007747

**Published:** 2009-11-13

**Authors:** Greta Snellman, Håkan Melhus, Rolf Gedeborg, Sylvia Olofsson, Alicja Wolk, Nancy L. Pedersen, Karl Michaëlsson

**Affiliations:** 1 Department of Surgical Sciences, Section of Orthopaedics, University Hospital, Uppsala, Sweden; 2 Department of Medical Sciences, Section of Clinical Pharmacology, University Hospital, Uppsala, Sweden; 3 Uppsala Clinical Research Centre, University Hospital, Uppsala, Sweden; 4 Department of Surgical Sciences, Section of Anaesthesiology and Intensive Care, University Hospital, Uppsala, Sweden; 5 Department of Nutritional Epidemiology, Institute of Environmental Medicine, Karolinska Institute, Stockholm, Sweden; 6 Departments of Medical Epidemiology and Biostatistics, Karolinska Institute, Stockholm, Sweden; VU University Medical Center, and Center for Neurogenomics and Cognitive Research, VU University Medical Center and VU University, The Netherlands

## Abstract

**Background:**

Although environmental factors, mainly nutrition and UV-B radiation, have been considered major determinants of vitamin D status, they have only explained a modest proportion of the variation in serum 25-hydroxyvitamin D. We aimed to study the seasonal impact of genetic factors on serum 25-hydroxyvitamin D concentrations.

**Methodology/Principal findings:**

204 same-sex twins, aged 39–85 years and living at northern latitude 60°, were recruited from the Swedish Twin Registry. Serum 25-hydroxyvitamin D was analysed by high-pressure liquid chromatography and mass spectrometry. Genetic modelling techniques estimated the relative contributions of genetic, shared and individual-specific environmental factors to the variation in serum vitamin D. The average serum 25-hydroxyvitamin D concentration was 84.8 nmol/l (95% CI 81.0–88.6) but the seasonal variation was substantial, with 24.2 nmol/l (95% CI 16.3–32.2) lower values during the winter as compared to the summer season. Half of the variability in 25-hydroxyvitamin D during the summer season was attributed to genetic factors. In contrast, the winter season variation was largely attributable to shared environmental influences (72%; 95% CI 48–86%), i.e., solar altitude. Individual-specific environmental influences were found to explain one fourth of the variation in serum 25-hydroxyvitamin D independent of season.

**Conclusions/Significance:**

There exists a moderate genetic impact on serum vitamin D status during the summer season, probably through the skin synthesis of vitamin D. Further studies are warranted to identify the genes impacting on vitamin D status.

## Introduction

Vitamin D is unique among vitamins because it can be synthesised endogenously by skin exposure to UV-B radiation. Globally, sun exposure is the most important source of vitamin D [1]. At northern latitudes, however, dietary intake by natural food sources, food fortification or dietary supplements is also of importance because exposure to UV-B radiation is low during the winter season [2]. Relatively little is known about genetic influences on vitamin D levels and virtually nothing about seasonal differences in these effects.

The primary step in endogenous vitamin D synthesis is the formation of pre-vitamin D_3_ from activation of 7-dehydrocholesterol when the skin is exposed to UV-B. Pre-vitamin D_3_ and vitamin D_2_ and D_3_ from food are converted to 25-hydroxyvitamin D in the liver and thereafter to the metabolic active form 1,25-dihydroxyvitamin D in the kidney. Sufficient levels of 1,25-dihydroxyvitamin D have a negative feedback on the secretion of parathyroid hormone [3]. 25-hydroxyvitamin D can be stored in body fat and mobilised when needed and thereby avoiding deficiency during the winter season. Serum 25-hydroxyvitamin D is the indicator of vitamin D status.

Vitamin D deficiency is common, especially among people in defined risk groups, such as the elderly living in nursing homes, obese persons, those normally veiled and dark-skinned people [4], [5], [6], [7]. Deficiency is principally associated with osteoporosis because 1,25-dihydroxyvitamin D stimulates calcium absorption in the intestines and bone mineralisation [3]. Vitamin D and its physiological effects have been extensively investigated in both men and women, particularly in its relationship to osteoporosis [8], [9], [10]. The possibility of preventing low energy fractures with substitution has also been broadly studied but the results are inconsistent, although it is generally accepted that supplementation with vitamin D in combination with calcium significantly reduces the risk of low energy fractures among institutionalised elderly women [11]. Vitamin D deficiency is also negatively related to muscle strength and balance, which would increase the risk of injurious falls [12], [13]. Furthermore, it has been suggested that deficiency contribute to cancer occurrence, diabetes, cardiovascular disease, multiple sclerosis and overall mortality [14], [15], [16], [17], [18], [19].

In addition to dietary intake and sun exposure, it is not known whether there are any additional important determinants of vitamin D status. Only a modest proportion of the variation in vitamin D status is explainable by lifestyle habits alone [2], [20], [21]. Two studies have addressed the genetic impact on serum 25-hydroxyvitamin D. In a large study of female twins Hunter et al [22] found that genes explained 43% of the variation in serum 25-hydroxyvitamin D. Data from the Framingham Offspring Study [23] indicate that familial influences and genes together explained 29% of the variation in serum 25-hydroxyvitamin D. Season-specific heritability estimates and shared environmental influences, parts of the variation that can not be attributed to either individual UV-B radiation or nutrition, have not previously been estimated. In this twin study, in a high incidence area of osteoporotic fractures at northern latitudes, we therefore aimed to estimate the importance of genetic factors and shared environmental influences on vitamin D status by season.

## Materials and Methods

### Ethics Statement

The study protocol was approved by the local ethical committee of Uppsala and all participants gave written informed consent to participate in the study and to donate blood samples.

### Subjects

Participants were recruited from The Swedish Twin Registry. All intact like-sexed twin pairs born 1965 or earlier and living in the county of Uppsala were invited to participate. Uppsala County is located in central Sweden at northern latitude 60°. Totally, 172 twin pairs were found eligible and invited to participate. Of these, 102 twin pairs (59 female and 43 male), i.e. 204 subjects, accepted to participate in the study. All twins were Caucasians. No subjects were excluded. Zygosity information in the Swedish twin registry has high validity [24]. The age range for women was 40–85 years and for men 39–83 years. In accordance with previous studies at northern latitudes, the winter season was defined as November 1 through April 30 and the summer season as May 1 through October 31 [25]. Vitamin D cannot be synthesised between late autumn through April at high latitudes, and winter requirements are met from the vitamin D store that has accumulated the previous summer [26]. The investigation was performed during the winter season for 28 twin pairs and during the summer season for 74 pairs. Because there is an individual seasonal variation in vitamin D levels, we tried to examine both members of each pair within the same week. Sixty-one pairs were examined the same day. The median within pair difference in days between the examinations among pairs that were not examined the same day was 6 days (interquartile range 2 to 9 days), with a maximum of 17 days. Only seven twins reported the use of vitamin D supplements.

### Biochemical Analyses

Venous blood samples were collected after a 12-h overnight fasting, protected from light, centrifuged and stored at −80°C until analysis was performed. Determination of 25-hydroxyvitamin D_2_ and 25-hydroxyvitamin D_3_ in plasma with high-pressure liquid chromatography (HPLC)- atmospheric pressure chemical ionisation (APCI)- mass spectrometry (MS) was determined at Vitas, Oslo, Norway. One hundred and fifty µL of human plasma was diluted with 450 µL 2-propanol containing butylated hydroxytolouene as an antioxidant. After thorough mixing (15 min) and centrifugation (10 min, 4000 g at 10°C), an aliquot of 35 µL was injected from the supernatant into the HPLC system. HPLC was performed with a HP 1100 liquid chromatograph (Agilent Technologies, Palo Alta, CA, USA) interfaced by atmospheric pressure chemical ionisation to a HP mass spectrometric detector operated in single ion monitoring mode. Vitamin D analogues were separated on a 4.6 mm×50 mm reversed phase column with 1.8 µM particles. The column temperature was 80°C. A two-point calibration curve was made from an analysis of albumin solution enriched with known vitamin D concentration. Recovery is 95%, the method is linear from 5–400 nM at least and the limit of detection is 1–4 nM. The CV is 7.6% (47.8 nM) and 6.92% (83.0 nM).

Levels of 1,25-dihydroxyvitamin D in serum were measured with Gamma-B 1,25–dihydroxyvitamin D RIA (IDS, Boldon, England). Precision for this intra-assay analysis is 6.3% CV; the inter-assay analysis 9.5% CV and the sensitivity is <11 pmol/L. To ascertain analytic quality all standards, controls and samples were analysed in duplicate and all duplicates with a CV >10% were reanalysed. The control samples provided by the manufacturer were within the recommended range.

### Statistical Analyses

Analyses of the relative importance of genes and environments for a phenotype can be carried out with traditional quantitative genetic methods well developed for twin studies [27]. The classical twin method is based on the knowledge that MZ twins share 100% of their genome, whereas DZ twins share, on average, 50% of their segregating genes. A higher intraclass correlation coefficient for values within MZ twin pairs than within DZ twin pairs provides a first indication of genetic influences on a trait. Information concerning shared genetic and environmental influences is best estimated by structural equation modelling techniques that fit models over all types of twins to best describe the causes of variation in a phenotype. The total variance in the trait can be partitioned into genetic variance (A), shared environmental variance [including shared (familial) environmental variance, C] and non-shared (unique environmental) variance (E), which also include measurement error. To estimate the parameters of interest the equation for one of the twins can be written as: 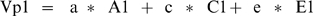
(1)


Vp1, A1, C1 and E1 are the total phenotypic variance, additive genes, shared environments and unique environments, respectively, for the first twin in the pair. A similar equation can be written for the second twin. The theoretical expectations for variance and covariance within twin pairs can be described with the following equations:
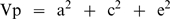
(2)

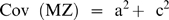
(3)

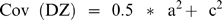
(4)


The parameters a, c and e can then be estimated with maximum likelihood methods and the relative importance of genes and environments can be evaluated. Heritability (A), the relative importance of genetic influences for variation in a trait, is defined as genetic variance (a^2^) divided by the total phenotypic variance. All genetic model fitting procedures were carried out using the computer software Mx [28], script rawVC1a.mx, and all estimates are adjusted for age (as a continuous variable) at the time of the investigation. However, only minor differences of the estimates were obtained with crude compared with the presented age-adjusted results. Likewise, additional adjustment of vitamin D supplement use only marginally affected our estimates and therefore these results are not presented.

The significance of genetic and shared environmental influences was tested by separately removing them from the full model, i.e. the ACE model was compared to the AE and the CE model. The difference of minus two times the log-likelihood (-2lnL) for a reduced model and that of the full model is approximately χ2 distributed with degrees of freedom (df) equal to the difference of the number of estimated parameters in the full model and the number of estimated parameters in the reduced model. If the difference is significant, the reduced model fits less well and the parameter that was dropped is necessary. The model with the lowest -2lnL was considered to have best model fit. There is substantial individual seasonal variation in serum 25-hydroxyvitamin D concentration [26]. We therefore hypothesised that the genetic influences on the concentrations were differentially affected by the season in which the samples were collected. Accordingly, results by season are presented separately. The seasonal difference in genetic influence was formally tested by comparing the sum of -2lnL for the winter and summer season models with the -2lnL of a model that simultaneously included both seasons. A P-value <0.05 was considered significant.

## Results

Descriptive characteristics as a function of season are summarised in Table 1. The average total 25-hydroxyvitamin D concentration was 84.8 nmol/L (95% CI 81.0–88.6); however, 17 twins (8%) had a concentration below 50 nmol/L. There were no differences in plasma PTH between twins with 25-hydroxyvitamin D concentrations above this cut-off (mean PTH 1.93 pmol/L) compared to below (mean PTH 1.95 pmol/L). Only one individual had a 25-hydroxyvitamin D concentration below 25 nmol/L. This twin had a higher serum PTH of 2.8 pmol/L. Only 40 twins (20%) had detectable 25-hydroxyvitamin D_2_. The mean serum 25-hydroxyvitamin D_2_ concentration in these twins was 7.8 nmol/L and the mean 25-hydroxyvitamin D_3_ concentration 79.2 nmol/L, giving a mean of the total 25-hydroxyvitamin D of 86.9 nmol/L.

**Table 1:** Characteristics of the twins as a function of season.

	Total (n = 204)	Summer season (n = 148)	Winter season (n = 56)
	Mean (SD)	Mean (SD)	Mean (SD)
S-25-hydroxyvitamin D_2+**3**_ (nmol/L)	84.8 (27.4)	91.2 (26.2)	67.2 (22.8)
S-25-hydroxyvitamin D_3_ (nmol/L)	83.3 (27.4)	89.6 (26.3)	65.9 (22.8)
S-1,25-dihydroxyvitamin D (pmol/L)	117.4 (43.5)	123.8 (45.2)	99.7 (32.7)
Age (years)	57.5 (9.7)	56.3 (7.1)	60.6 (14.3)
Weight (kg)	74.0 (12.2)	75.0 (12.5)	71.0 (10.7)
Height (cm)	170.5 (9.7)	171.6 (9.6)	167.5 (9.4)
Body mass index (kg/m^2^)	25.4 (3.2)	25.4 (3.1)	25.3 (3.4)
P-PTH (pmol/L)	1.9 (0.9)	2.0 (1.0)	1.9 (0.9)

**Figure 1:**
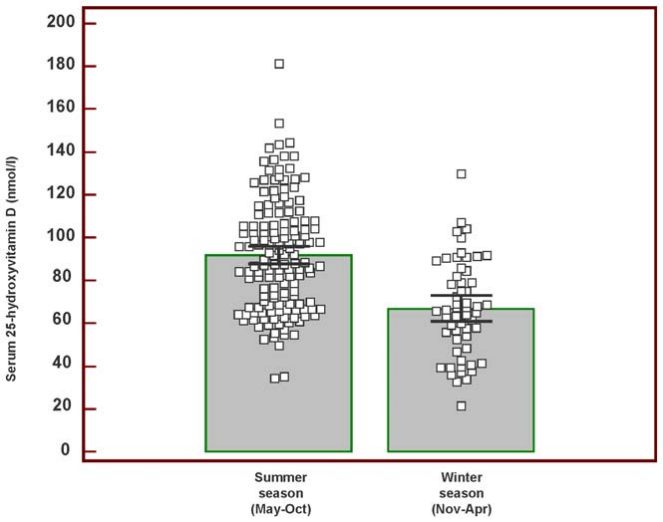
The distribution in serum 25-hydroxyvitamin D by season. Each value is designated by a box. The height of the wide grey bars indicates mean values and the error bars display 95% confidence intervals for the means.

The individual total serum 25-hydroxyvitamin D concentrations by season are displayed in Figure 1. The serum concentrations were significantly higher during the summer season. The difference between mean summer and mean winter season concentrations was 24.2 nmol/L (95% CI 16.3–32.2), with a larger variance during the summer season (687 and 520, respectively). We found no gender differences in serum 25-hydroxyvitamin D concentrations. During the summer season, the male to female difference was 0.9 nmol/L (95% CI −7.8–9.4, p = 0.84) and during the winter season 3.2 nmol/L (95% CI −10.8–17.2, p = 0.65).

There was a tendency of stronger correlation for serum 25-hydroxyvitamin D within monozygotic twin pairs (intraclass correlation coefficient (ICC) 0.73; 95% CI 0.57–0.84) than among dizygotic twins (ICC 0.61; 95% CI 0.41–0.75). However, the difference in pair-wise resemblance among monozygotic compared to dizygotic twins was no longer evident if we restricted the analysis to twins whose blood was collected during the winter season. During this period, the correlation was 0.71 (95% CI 0.39–0.88) among monozygotic twins and 0.73 (95% CI 0.31–0.91) among dizygotic twins. The corresponding values for twins examined during the summer season were 0.64 (95% CI 0.39–0.81) and 0.51 (95% CI 0.27–0.70), respectively.

The average heritability and environmental influences on total serum 25-hydroxyvitamin D independent of season are presented in Table 2. There was a modest heritability (A) of serum 25-hydroxyvitamin D and an equally strong shared environmental influence (C). The non-shared (individual-specific) environmental influence (E) on variation in serum 25-hydroxyvitamin D contributed to approximately one fourth of the variation. Similar results were obtained if we only analysed 25-hydroxyvitamin D_3_ instead of total 25-hydroxyvitamin D. We found significantly worse model fits for the AE and the CE models compared to the complete ACE model, confirming that both A and C are significant.

**Table 2:** The age-adjusted genetic, shared and non-shared (individual specific) environmental influences on variance of serum vitamin D levels (95% CI) among 204 Swedish adult twins.

Model	Proportion of variance explained by genetic influence (A)	Proportion of variance explained by shared environmental influence (C)	Proportion of variance explained by non-shared environmental influence (E)	−2 log-likelihood	P value for difference from ACE model
**Total 25-hydroxyvitamin D**					
ACE	0.39 (0.32–0.75)	0.38 (0.04–0.42)	0.23 (0.15–0.36)	1856.10	Ref
AE	0.78 (0.66–0.85)	-	0.22 (0.15–0.34)	1860.61	0.03
CE	-	0.66 (0.54–0.76)	0.34 (0.24–0.46)	1859.95	0.05
**25-hydroxyvitamin D_3_**					
ACE	0.39 (0.32–0.43)	0.38 (0.32–0.42)	0.23 (0.15–0.35)	1854.53	Ref
AE	0.78 (0.66–0.85)	-	0.22 (0.15–0.34)	1858.98	0.04
CE	-	0.66 (0.54–0.76)	0.34 (0.24–0.46)	1858.42	0.05

However, there was a substantially improved overall model fit when our data were presented separately by season rather than constrained to be equal across seasons (χ2 value 28.7, 6 df, p<0.0001). Accordingly, a considerable seasonal difference in 25-hydroxyvitamin D heritability was seen (Table 3). Half of the total variance during the summer season was explained by the genetic variance compared to low heritability estimates during the winter season. In contrast, there was a substantial impact of C during the winter season. The CE model was the most parsimonious model for the winter season, both for total 25-hydroxyvitamin D and 25-hydroxyvitamin D_3_. C was estimated to 0.72 (95% CI 0.48–0.86) for total 25-hydroxyvitamin D and 0.73 (95% CI 0.51–0.86) for 25-hydroxyvitamin D_3_. Non-shared (individual-specific) environmental influences were found to explain only approximately one fourth of the variation in serum 25-hydroxyvitamin D independent of season.

**Table 3:** The age-adjusted genetic, shared and non-shared (individual specific) environmental influences on variance of total serum 25-hydroxyvitamin D and 25-hydroxyvitamin D_3_ levels (95% CI) during summer and winter seasons among Swedish adult twins.

Model	Proportion of variance explained by genetic influence (A)	Proportion of variance explained by shared environmental influence (C)	Proportion of variance explained by non-shared environmental influence (E)	−2 log-likelihood	P value for difference from ACE model
**Summer season n = 148**					
**Total 25-hydroxyvitamin D**					
ACE	0.48 (0.00–0.85)	0.25 (0.00–0.64)	0.26 (0.14–0.51)	1342.48	Ref
AE	0.74 (0.56–0.84)	-	0.26 (0.16–0.44)	1344.22	0.19
CE	-	0.57 (0.39–0.70)	0.43 (0.30–0.61)	1345.46	0.08
**25-hydroxyvitamin D_3_**					
ACE	0.48 (0.00–0.85)	0.26 (0.00–0.64)	0.26 (0.14–0.50)	1342.83	Ref
AE	0.74 (0.57–0.85)	-	0.26 (0.15–0.43)	1344.46	0.20
CE	-	0.57 (0.40–0.71)	0.43 (0.29–0.60)	1345.92	0.08
**Winter season n = 56**					
**Total 25-hydroxyvitamin D**					
ACE	0.00 (0.00–0.77)	0.72 (0.00–0.86)	0.28 (0.14–0.52)	485.00	Ref
AE	0.73 (0.48–0.86)	-	0.27 (0.14–0.52)	489.22	0.04
CE	-	0.72 (0.48–0.86)	0.28 (0.14–0.52)	485.00	1.00
**25-hydroxyvitamin D_3_**					
ACE	0.00 (0.00–0.63)	0.73 (0.10–0.86)	0.27 (0.13–0.49)	484.10	Ref
AE	0.73 (0.47–0.86)	-	0.27 (0.14–0.53)	489.15	0.02
CE	-	0.73 (0.51–0.86)	0.27 (0.14–0.49)	484.10	1.00

## Discussion

The results of our twin study indicate a moderate genetic impact on vitamin D status in the summer season and a predominantly shared environmental influence during the winter season, presumably due to solar altitude. We are not aware of any previous study that has addressed these issues. During the winter season when UV-B radiation is minimal at latitude 60° north and the cutaneous synthesis of pre-vitamin D is non-existent [26], the heritability was undetectable. Finding decreased vitamin D concentrations in winter compared to summer was expected and is most likely due to the low vitamin D_3_ synthesis in the skin during the winter. Theoretically, genetic factors could be important at several levels, such as the capacity to synthesise vitamin D in the skin, to assimilate vitamin D from the diet and to store the vitamin in body fat (which is used as a reservoir during the winter). Assuming that dietary intake and body fat mass are relatively constant throughout the year [29], it seems reasonable to conclude that the key genetic influence is on the cutaneous synthesis of vitamin D.

Seasonal variation in UV-B radiation that cannot be referred to individual specific factors (such as time spent in southern altitudes or indoor tanning facilities) is included in the shared environmental factor. In our study, with participants who had a low consumption of vitamin D supplements, the individual specific variation in sun exposure and dietary intake, reflected by individual environmental influences, was stable across season and contributed to approximately one fourth of the variance in vitamin D status. Earlier studies have addressed the importance of sun exposure, diet and supplements on vitamin D status. Burgaz et al [2] found that spending a week in a sunny southern country during wintertime increased the vitamin D concentration among Swedish women with 16% and 17% among those who used dietary supplements regularly. Brot et al [20] studied the correlation between vitamin D status, sun exposure and dietary intake among Danish women. Active sunbathing increased vitamin D concentrations by 27% and dietary intake contributed to 9% of the variation in serum concentrations. Nevertheless, in total these non-shared (individual specific) environmental influences contributed to 13% of the variation in serum 25-hydroxyvitamin D [2]. Even if this is an attenuated estimate because of measurement errors of the exposures, it is in accordance with our results, i.e. individual lifestyle habits do not explain more than a modest proportion of the variation in serum 25-hydroxyvitamin D.

Contrary to what is expected, many studies have come to the conclusion that vitamin D concentrations are generally higher among people in northern Europe than among people in southern Europe [30], [31]. Our average serum 25-hydroxyvitamin D levels are in line with the earlier Swedish values estimated in the MORE study [30]. These values were, independent of season, approximately 30% higher than the average among people from central and southern Europe. The results have been explained by a diet containing more vitamin D-fortified foods, lighter skin and wearing lighter clothing when being outdoors during the summer [30], [31]. Our results indicate that our genes, as well as environmental factors, contribute to our vitamin D status. Higher vitamin D concentrations in northern countries may have a genetic basis.

This study has the advantage of being a twin study, a perfect natural experiment when studying the relative importance of environmental and genetic influences [32]. The study was population-based. The participants, all community-dwelling white men and women, had a homogenous ancestry because all were born in Sweden. There is presently no widely accepted definition of vitamin D insufficiency, but several authors have suggested that the serum concentration of 25-hydroxyvitamin D should exceed 50 nmol/L to prevent modifiable bone loss [5], [33]. Some studies suggest that PTH levels plateau and are at the optimal concentration when 25-hydroxyvitamin D is above 80 nmol/L, thus avoiding calcium resorption from bone and a decrease in muscle strength [25], [34]. Nevertheless, with a 50 nmol/L cut-off, only 8% of our twins were classified as vitamin D insufficient. A considerably larger proportion of persons in other countries at high latitudes have been found to be vitamin D insufficient [20], [29]. The discrepancy might be explained by different analytical methods of assaying serum 25-hydroxyvitamin D but also by vitamin D fortification of low-fat dairy products in Sweden. 25-hydroxyvitamin D concentrations can be measured by several methods, including HPLC, competitive protein binding assay (CPBA), radioimmunoassay (RIA), enzyme immunoassay (EIA) or automated chemiluminescence protein-binding assay (CLPBA). RIA, EIA and CLPBA are preferred in many laboratories and hospitals because of their simplicity and rapidity. However, these methods require high quality control to ensure reliable results and variability and inconsistencies are known problems [35], [36]. The RIA method was used in two earlier genetic studies [22], [23] of serum 25-hydroxyvitamin D. In both, a modest heritability of 25-hydroxyvitamin D was concluded but the seasonal genetic influence was not determined. HPLC is the most reliable method for determining 25-hydroxyvitamin D and is considered the gold standard for the validation of serum 25-hydroxyvitamin D measurement [37]. This method can discriminate between serum 25-hydroxyvitamin D_3_ and 25-hydroxyvitamin D_2_. The HPLC methods have recently been improved by the addition of mass spectrometry (MS) [37]. We have therefore chosen to measure 25-hydroxyvitamin D with a highly sensitive HPLC-MS method in this study.

Our study also has limitations. We examined the heritability of serum vitamin D in only white community-dwelling, mostly vitamin D replete participants living at northern latitude. Thus, we cannot extrapolate our results to groups with other pigmentation characteristics or to institutionalised subjects. We had the possibility to include only 204 twins that were further divided into subgroups. A larger sample would have been preferred. Fewer twins were tested during the winter season, which has the limitation of providing less stable results. Nevertheless, we found no overlap in the confidence intervals of the estimates in the most parsimonious models during winter time (CE model) and there was also a considerable improved total model fit (p<0.0001) when the data was presented by season. Our study had also the conceivable limitation of an unequal seasonal sampling distribution of the twins; fewer twins were allocated during the most sun deprived part of the year. Therefore, most twins included during our defined winter season were examined in March and April, which are in fact the nadir months of serum vitamin D status at our latitude [38]. This, which at first could be viewed as a limitation, is in fact therefore an advantage since early winter 25-hydroxyvitamin D values are much more influenced by vitamin D stores from the sunny season. We did not assess the underlying causes of the non-shared environmental influences, such as diet, use of supplements and individual UV-B radiation but only seven of our twins reported the use of vitamin D supplement. However, the relative contribution to vitamin D status of each of these lifestyle habits has previously been assessed by us [2]. Although not all eligible twins participated, our distribution of serum vitamin D was similar to that in the MORE study [30]. 25-hydroxyvitamin D_2_ has an origin from vegetable food. Supplements do not normally contain this vitamin D metabolite in Sweden. Only a small proportion of total 25-hydroxyvitamin D among our participants was 25-hydroxyvitamin D_2_, which can explain the small differences in our heritability estimates between total serum 25-hydroxyvitamin D and 25-hydroxyvitamin D_3_.

In conclusion, our results indicate that approximately half of the variation in serum vitamin D status during the summer season is explained by genetic factors. The winter season variation in serum levels can mainly be explained by shared environmental influences, i.e. solar altitude. These findings suggest that genetic factors play a major role in the generation of vitamin D in the skin and should encourage further research aiming at the identification of these factors.
