# Correction: Seasonal Genetic Influence on Serum 25-Hydroxyvitamin D Levels: A Twin Study

**DOI:** 10.1371/annotation/e85fe043-b072-422d-acd5-9d27f02390b3

**Published:** 2010-09-29

**Authors:** Greta Snellman, Håkan Melhus, Rolf Gedeborg, Sylvia Olofsson, Alicja Wolk, Nancy L. Pedersen, Karl Michaëlsson

University Hospital, Uppsala, Sweden is incorrectly used in the affiliations. It should be replaced with Uppsala University, Uppsala, Sweden. 

